# Methane limit LPS-induced NF-κB/MAPKs signal in macrophages and suppress immune response in mice by enhancing PI3K/AKT/GSK-3β-mediated IL-10 expression

**DOI:** 10.1038/srep29359

**Published:** 2016-07-11

**Authors:** Xu Zhang, Na Li, Han Shao, Yan Meng, Liping Wang, Qian Wu, Ying Yao, Jinbao Li, Jinjun Bian, Yan Zhang, Xiaoming Deng

**Affiliations:** 1 Department of Anesthesiology and Intensive Care, Changhai Hospital, The Second Military Medical University, Shanghai, 200433, China (mainland).; 2 Jiangsu Province Key Laboratory of Anesthesiology, Xuzhou Medical College, Xuzhou, 221004, Jiangsu, China; 3 Jiangsu Province Key Laboratory of Anesthesia and Analgesia Application Technology, Xuzhou, 221004, Jiangsu, China; 4 Department of Anesthesiology, General Hospital of Fuzhou Army Region, Fuzhou, 350025, China

## Abstract

Inflammatory diseases such as sepsis and autoimmune colitis, characterized by an overwhelming activation of the immune system and the counteracting anti-inflammatory response, remain a major health problem in worldwide. Emerging evidence suggests that methane have a protective effect on many animal models, like ischaemia reperfusion injury and diabetes-associated diseases. Whether methane could modulating inflammatory diseases remains largely unknown. Here we show that methane-rich saline (MS) *ip* treatment (16 ml/kg) alleviated endotoxin shock, bacteria-induced sepsis and dextran-sulfate-sodium-induced colitis in mice via decreased production of TNF-α and IL-6. In MS-treated macrophages, LPS-induced activation of NF-κb/MAPKs was attenuated. Interestingly, MS treatment significantly elevated the levels of IL-10 both *in vitro* and *in vivo*. Neutralization of IL-10 abrogated the therapeutic effect of MS. Moreover, anti-IL10 blockade partially restored the MS-mediated attenuation of NF-κb/MAPKs phosphorylation. We further found that MS resulted in markedly enhanced phosphorylation of GSK-3β and AKT, which both mediate the release of Il-10. Additionally, inhibition of PI3K attenuated MS-mediated p-GSK-3β and IL-10 production and reversed the suppressed activation of NF-κb/ MAPKs in response to LPS. Our results reveal a novel effect and mechanisms of methane and support the potential value of MS as a therapeutic approach in innate inflammatory diseases.

Innate immune responses provide the first line of host defence against invading pathogens. Pathogen-associated molecular patterns (PAMPs) recognition by the innate immune system largely relies on Toll-like receptors (TLRs), which are present on innate immune cells, including macrophages and dendritic cells. Among the identified TLRs, TLR4 was the first one to have its ligand identified as lipopolysaccharide (LPS), which are found in the cell wall of Gram-negative bacteria^1^. The recognition of PAMPs by TLRs leads to the activation of signalling through a MyD88- or TRIF-dependent pathway^2^. Apart from TLR3, all TLRs signal through MyD88, which leads to the activation and nuclear translocation of nuclear factor-κb (NF-κb) and mitogen-activated protein kinase (MAPKs). NF-κb and MAPKs signalling leads to AP-1 activation, resulting in the transcription of pro-inflammatory genes, including pro-inflammatory cytokines such as tumour necrosis factor (TNF-α), interleukin-6 (IL-6), and IL-12 p40, as well as many chemokines^3^,^4^.

Although TLR-induced inflammatory responses are required for pathogen clearance, inappropriate TLR responses may contribute to the pathogenesis of acute and chronic inflammation. Such inappropriate TLR responses are most apparent with TNF-α and IL-6, which are involved in the acute inflammation observed in endotoxin shock, sepsis and several autoimmune diseases, such as inflammatory bowel disease (IBD), rheumatoid arthritis (RA) and systemic lupus erythematosus (SLE)^5^,^6^,^7^.

On the contrary, anti-inflammatory cytokines, such as IL-10, have been shown to inhibit the LPS-induced production of pro-inflammatory cytokines^8^. IL-10 is a potent anti-inflammatory cytokine produced by macrophages and DCs upon TLR signalling to inhibit inflammatory responses through signal transducer and activator of transcription 3 (STAT3)^8^. Dysregulated IL-10 production has been implicated in inflammatory diseases^9^,^10^. Strict regulation of the balance between the production of IL-10 and pro-inflammatory cytokines during infection is necessary to efficiently eliminate invading pathogens while avoiding harmful immunopathology. Because macrophages are both the major producers and the targets of IL-10 in the innate immune response, preventing the harmful effects that are associated with the production of pro-inflammatory cytokines and IL-10 in macrophages may offer new therapeutic approaches for the treatment of acute and chronic inflammation.

Methane, the simplest aliphatic hydrocarbon, is known as fuel and a potent greenhouse gas. Interestingly, it can also be synthesized biologically in the colon^11^ or in the mitochondria of rat livers under hypoxic conditions^12^,^13^. Methane production has been shown to be associated with intestinal diseases, such as constipation-predominant irritable bowel syndrome (C-IBS), diverticulosis, and colon cancer^14^,^15^,^16^,^17^. Studies have revealed that methane can exert protective effects on intestinal^18^, myocardial^19^, hepatic^20^, abdominal skin flaps injury caused by ischaemia and reperfusion^21^, and diabetic retinopathy^22^ in rat or dog models. These studies indicate that methane might be a promising potential therapy in ischaemia and reperfusion disease and diabetes-associated disease.

However, the limited numbers of studies using rat models have focused mainly on ischaemia and reperfusion models. Thus, whether methane has a protective role the innate immune inflammatory mouse model is largely unknown. Mechanistically, published reports have revealed that methane exerts its protective effect via its anti-oxidative, anti-inflammatory, and anti-apoptotic activities^18^,^19^,^20^,^21^,^22^. Although studies have shown that methane treatment can significantly reduce the level of TNF-α and IL-1β in an ischaemia and reperfusion rat model, how methane suppresses the production of pro-inflammatory cytokines in this models remains unclear.

Here, we demonstrate that methane-rich saline (MS) treatment can protect mice from LPS-induced endotoxin shock, bacteria-induced sepsis and DSS-induced colitis by suppressing TNF-α and IL-6 production. Mechanistically, MS attenuated the phosphorylation of NF-κb, JNK, ERK and P38MAPK in LPS-stimulated macrophages in an IL-10-dependent manner via enhanced GSK-3β phosphorylation. We also show that the enhanced activation of PI3K/AKT signalling is involved in GSK-3β activation induced by MS. Our study expands the scope of methane therapy and provides new mechanistic insight into the anti-inflammatory effect of MS.

## Results

### Methane inhibits TNF-α and IL-6 production in LPS-induced peritoneal macrophages and bone marrow-derived macrophages of mice

**Figure 1:**
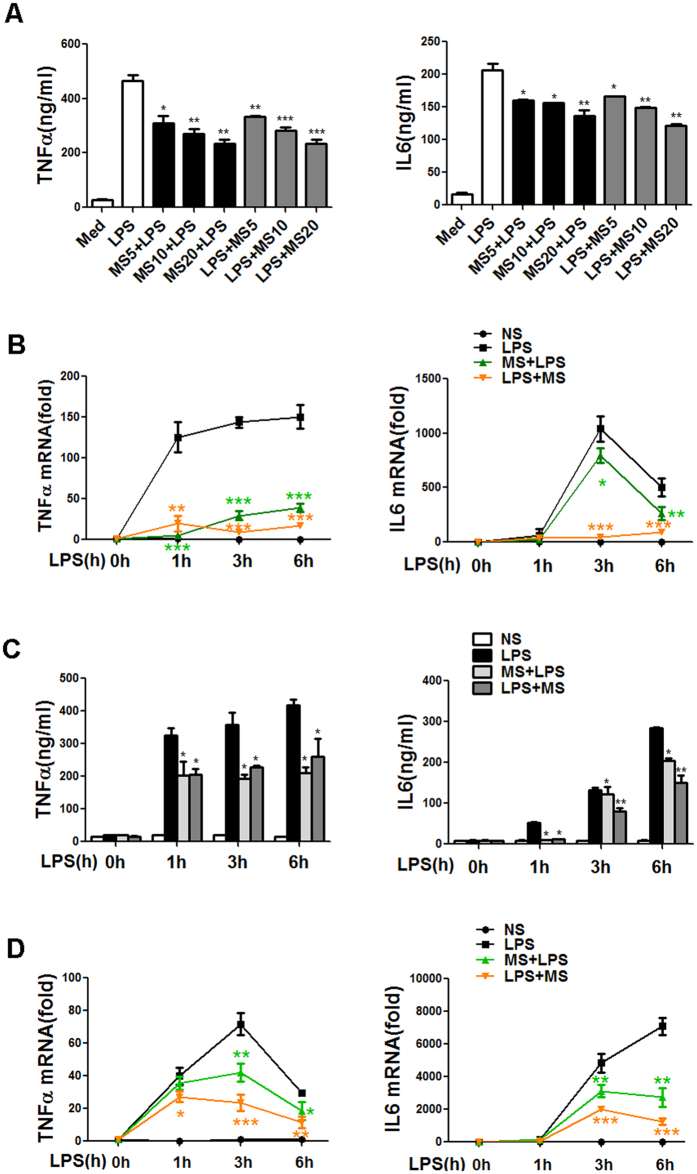
MS suppresses the production of pro-inflammatory cytokines in LPS stimulated peritoneal macrophages (PMs) and bone marrow-derived macrophages (BMDMs) of mice. PMs (**A,B**) or BMDMs (**C,D**) were treated with LPS (100 ng/ml) for 6 h and the media were collected and analysed for the TNF-α and IL-6 level by ELISAs (**A,C**). For the protein expression analysis, MS was added 30 min before or after LPS stimulation at the indicated concentrations. For the mRNA expression analysis, MS was added 10 μl/ml for pre-treatment and post-treatment. The mRNA expression was quantified by Q-PCR at the indicated time points (**B,D**). Data are expressed as the mean ± SD of at least three independent experiments. Unpaired Student’s t-test, *P < 0.05; **P < 0.01; ***P < 0.001 compared with the LPS-induced control.

As the key components in innate immunity, macrophages produce inflammatory cytokines and present foreign antigens upon TLR ligation. TNF-α and IL-6 are two of the major inflammatory mediators that contribute to the pathogenesis of acute and chronic inflammation. To investigate the role of MS in LPS-induced inflammatory cytokine production, the TNF-α and IL-6 levels were measured in LPS-stimulated mouse peritoneal macrophages using ELISAs. We found that 6 h of exposure to LPS led to the increased levels of TNF-α and IL-6. MS pre-treatment or post-treatment both inhibit the expression of LPS-induced TNF-α and IL-6 protein in a dose-dependent manner (Fig. 1A). Next, we investigated whether MS inhibited TNF-α and IL-6 expression at the mRNA level (Fig. 1B). Our data showed that MS pre-treatment and post-treatment both significantly suppressed the LPS-induced mRNA upregulation of both cytokines as early as 1 h after LPS administration. Especially for IL-6, as its mRNA level was reduced by MS post-treatment by approximately 83% and 95% at the 3 h and 6 h after LPS stimulation, respectively. It seems that MS post-treatment exert a stronger inhibitory effect than pre-treatment.

We next tested the anti-inflammatory effect of MS in bone marrow-derived macrophages and obtained similar results. Remarkably, the inhibitory effect of MS on the production of TNF-α and IL-6 upon LPS engagement was observed at the protein level (Fig. 1C) and at the mRNA level (Fig. 1D). These data suggest that MS suppresses the production of pro-inflammatory cytokines upon LPS exposure in mouse macrophages.

### MS protects against endotoxin shock and bacterial challenge in mice

**Figure 2:**
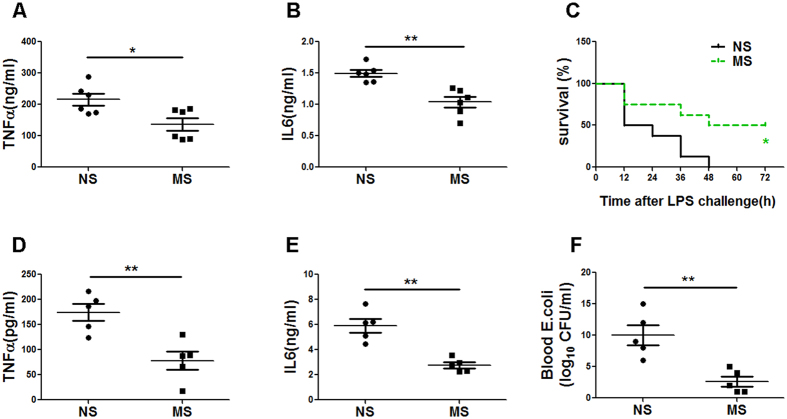
MS protects mice from challenge with LPS and bacteria-induced sepsis. (**A,B**) ELISA of TNF-α and IL-6 in the serum after LPS challenge (10 mg/kg body). For MS post-treatment, the mice were injected with saline or MS at a dose of 16 ml/kg intraperitoneally 0.5 h after LPS challenge. (n = 6 per group). (**C**) The survival of the mice receiving MS treatment or control mice given an i.p. injection of LPS (15 mg/kg body weight) is shown. (n = 8 per group. *P < 0.05, Wilcoxon test). (**D,E**) ELISA of TNF-α (**D**) and IL-6 (**E**) in the serum of mice 3 h after i.p. infection with 1 × 10^7^ *E. coli* 0111:B4. (n = 5 per group). (**F**) Analysis of the bacterial loads in the blood of mice receiving MS treatment or control mice 5 h after i.p. infection. (n = 5 per group). The results in (**A,B,D-F**) are presented as the mean ± SD. *P < 0.05; **P < 0.01; ***P < 0.001 (Student’s t-test, analysis of variance compared with the NS control groups).

To assess the effect of MS in the TLR4-triggered innate immune response, we first challenged mice with LPS intraperitoneally, followed by 16 ml/kg MS or normal saline (NS) treatment. Blood samples were collected and analysed for TNF-α and IL-6 levels 6 h later. Consistent with the *in vitro* observations, MS-treated mice produced significantly less TNF-α and IL-6 than the control mice in response to LPS (Fig. 2A,B). Accordingly, these mice exhibited prolonged survival after lethal LPS challenge compared with control mice (Fig. 2C). These data show that MS suppresses TLR4-triggered inflammatory responses in mice during endotoxin shock.

Next, we assessed the role of MS in the host innate response to pathogen infection by challenging the mice with intact Gram-negative *E. coli*. We found that the levels of TNF-α and IL-6 in the sera of MS-treated mice were significantly lower than those of the control mice (Fig. 2D,E), and the MS-treated mice had a lower load of *E. coli* bacteria in the blood (Fig. 2F), consistent with the published finding that pro-inflammatory cytokines promote the dissemination of *E. coli*^23^. Another group in our laboratory also observed the protective effect of MS on CLP mice (data not shown). These data indicate that MS attenuates the acute innate inflammatory response of the host and protects mice from endotoxin shock and bacteria-induced sepsis.

### MS suppresses dextran sulfate sodium-induced colitis

**Figure 3:**
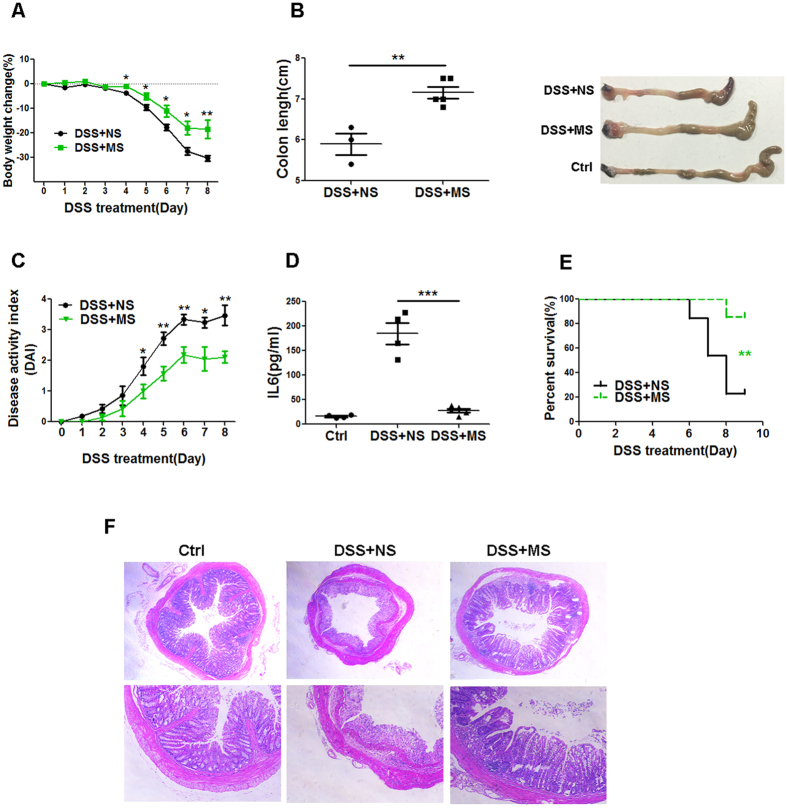
MS inhibits inflammation in DSS-induced colitis. DSS was administered ad libitum in distilled water at a 3% concentration for 5 d followed by normal drinking water. For MS treatment, the mice were i.p. administrated with saline or MS (16 ml/kg body weight) on the day 2, day 3 and day 4 during the DSS administration. The body weights were determined on the indicated days (**A**), (n = 7 per group). On day 8 after the DSS treatment, the colon length was measured (**B**) and examined by H&E staining (**F**), and the IL-6 levels in the serum were measured by ELISA (**D**). The disease activity index (DAI) (**C**) and survival (**E**), (In = 7 per group, **P < 0.01, Wilcoxon test) were evaluated daily. The results in (**A–D**) are presented as the mean ± SD. *P < 0.05; **P < 0.01; ***P < 0.001 (Student’s t-test, analysis of variance, compared with the NS control groups). The experiments were performed at least twice, yielding similar results.

We next examined whether MS exerts a protective effect in control of inflammatory response. We used a mouse model of colitis induced by dextran sulfate sodium (DSS), a typical inflammatory model with many similarities to human inflammatory bowel diseases (IBD) that primarily depends on the innate immune system. Pro-inflammatory cytokines such as IL-6 have been implicated in mediating inflammatory responses in DSS induced colitis^5^. As expected, we found that compared with control mice, MS-treated mice showed attenuated colon inflammation, as evidenced by a slower decrease in the body weight (Fig. 3A), less shortening of the colon length (Fig. 3B), a reduced disease activity index (DAI) (Fig. 3C), a significantly lower level of IL-6 in the sera (Fig. 3D), the infiltration of fewer inflammatory cells, and the less disruption of mucosal structures in histological analyses of the colons (Fig. 3F). Consistent with these improvements, these mice exhibited prolonged survival (Fig. 3E). Notably, the IL-6 level in MS-treated mice decreased by approximately 85% compared with control mice, indicating the potent inhibitory effect of MS on the production of IL-6. Our data indicated that MS suppresses the development of inflammatory diseases such as IBD.

### Methane suppresses TLR4-induced NF-κb/MAPKs activation

**Figure 4:**
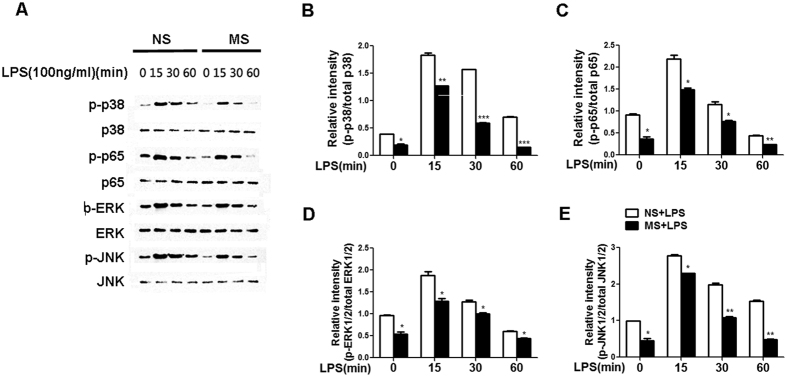
MS attenuates the phosphorylation of NF-κb and MAPKs. (**A**) Peritoneal macrophages were treated with 100 ng/ ml LPS for different times, MS (10 μl/ml) or NS was added 10 min after LPS stimulation, and the protein levels of the indicated molecules were examined by western blotting. (**B–E**) The results in A were quantified by determining the band intensity and calculated as the ratio of phosphorylated signalling molecules to total corresponding molecules. Data are presented as the mean ± SD of three repeats. *P < 0.05; **P < 0.01; ***P < 0.001 (Student’s t-test, analysis of variance, compared with the NS control).

Next, we sought to explore the underlying mechanism of the MS-mediated repression of inflammatory mediators in TLR-triggered immune responses. It is well known that the activation of the MAPK and NF-κb pathways is necessary for TLR-induced pro-inflammatory cytokine production. Thus, we investigated the possible effect of MS on the TLR-induced phosphorylation of MAPKs and NF-κb. As shown in Fig. 4, TLR4 triggering led to a rapid activation of P38MAPK, NF-κb, ERK and JNK at the 15 min and 30 min time points, and their activation decreased 60 min after LPS stimulation. By contrast, the activation of these molecules was significantly reduced at all indicated time points in the MS post-treatment group. We also observed a similar inhibitory effect of MS pre-treatment (15 min before LPS exposure) on the activation of these signalling molecules (data not shown). Our data indicate that MS treatment attenuates the activation of NF-κb and MAPKs induced by LPS, which might account for the decreased production of TNF-α and IL-6 upon TLR4 signalling in macrophages.

### MS promotes IL-10 production *in vitro* and *in vivo*

**Figure 5:**
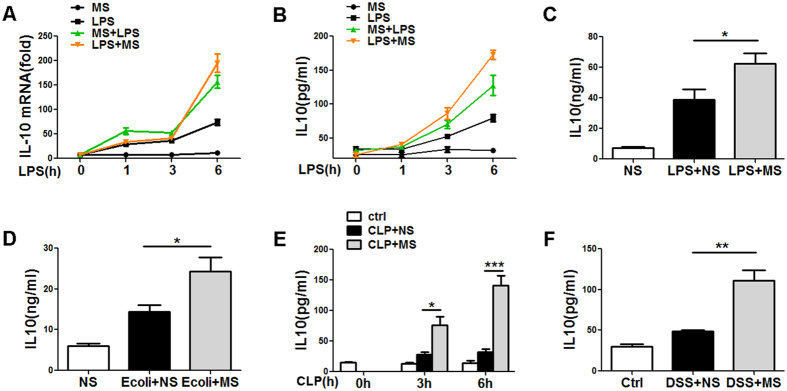
MS promotes IL-10 production in macrophages and *in vivo*. (**A,B**) Peritoneal macrophages were treated with 100 ng/ml LPS and MS (10 μl/ml) was given 30 min after LPS stimulation. The IL-10 levels were determined by Q-PCR (**A**) and ELISA (**B**) at the indicated time points. (**C**) ELISA of the serum IL-10 levels 6 h after LPS challenge in mice (intraperitoneal injection of LPS, 10 mg/kg body weight). For MS post-treatment, the mice were given MS (16 ml/kg body weight) or saline i.p. 30 min after LPS injection (n = 6). (**D**) The serum IL-10 levels 5 h after i.p. infection with 1 × 10^7^ *E. coli 0111:B4* followed by i.p.-injected MS (16 ml/kg body weight) or saline 30 min after *E. coli* challenge (n = 4). (**E**) The serum IL-10 level 6 h after CLP or in combination with MS post-treatment (16 ml/kg body weight) (n = 4). (**F**) ELISA of the serum IL-10 levels of MS and control mice (n = 5) at 6 h on day 1 after feeding with 3% DSS and i.p. injection with MS (16 ml/kg body weight). Data are presented as the mean ± SD of at least two repeats. *P < 0.05; **P < 0.01; ***P < 0.001 (Student’s t-test, analysis of variance, compared with the NS control).

Interleukin-10 (IL-10), a pleiotropic cytokine that limits inflammatory responses, is produced by many cell lines like macrophages, monocytes, and lymphocytes. The induction of IL-10 is critical for down-modulating the pro-inflammatory response and preventing tissue injury caused by excessive inflammation^9^,^24^. To explore the anti-inflammatory mechanism of MS, we analysed the IL-10 expression in LPS-stimulated macrophages and found that both pre-treatment and post-treatment with MS induced the significant upregulation of IL-10 at the protein (Fig. 5A) and mRNA levels (Fig. 5B). In agreement with our previous observation *in vitro*, it seems that post-treatment with MS 0.5 h after LPS exposure resulted in a higher level of expression of IL-10 than that observed with MS pre-treatment.

These *in vitro* results were further supported by analysis of IL-10 levels in mice following endotoxin shock (Fig. 5C), mice with bacteria-induced sepsis (Fig. 5D), CLP mice (Fig. 5E) and mice with DSS-induced colitis (Fig. 5F). In all four mouse models, MS treatment resulted in a significantly higher level of IL-10 compared with control mice. These data suggest that MS increases IL-10 production both in LPS-stimulated macrophages and in the four mouse models mentioned above.

### IL-10 is required for the anti-inflammatory effect of MS both *in vitro* and *in vivo*

**Figure 6:**
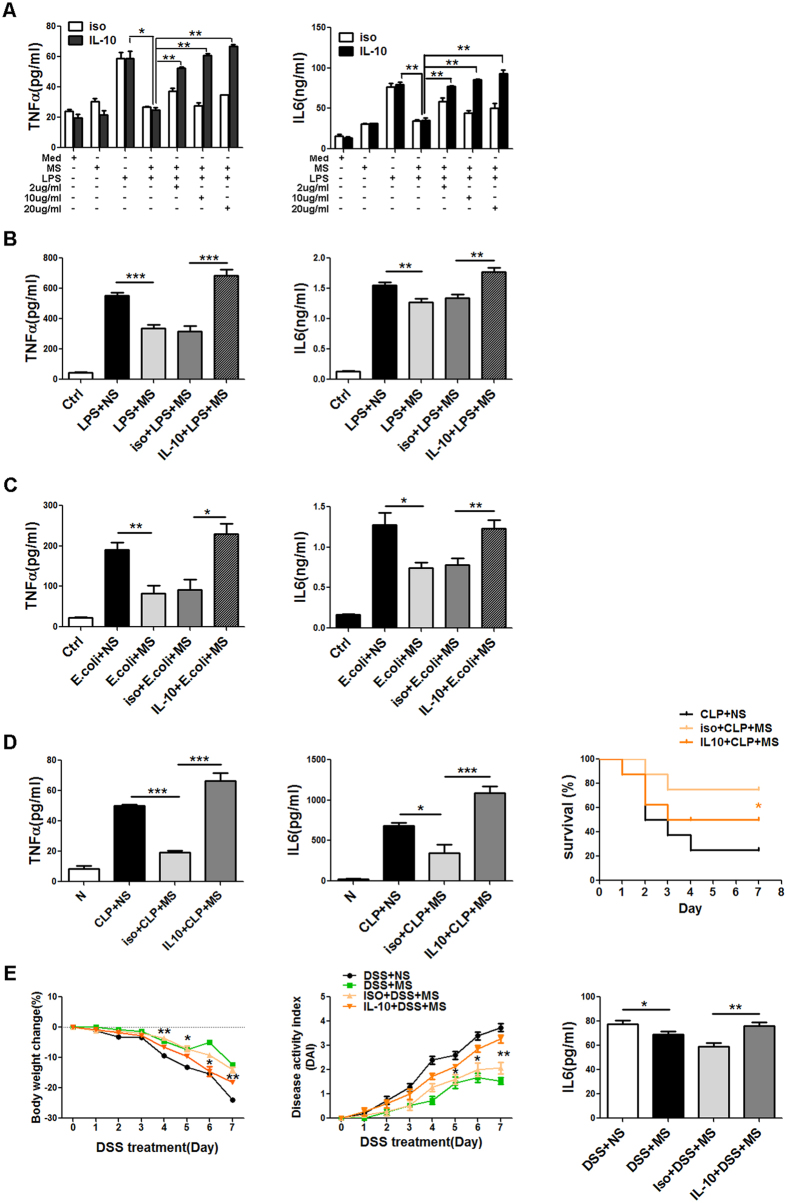
Blockade of IL-10 reverses the inhibitory effect of MS *in vitro* and *in vivo*. (**A**) Macrophages were pre-treated with different doses of isotype antibody or anti-IL-10 antibody for 1 h and stimulated with 100 ng/ ml LPS for 0.5 h followed by 10 μl/ml MS. 6 h after LPS challenge, the supernatants were collected and the TNF-α and IL-6 levels were analysed by ELISA. (**B–E**) The mice were intraperitoneally injected 40 μg/per mouse with isotype or anti-IL-10 antibody for 24 h and challenged with LPS (B, i.p. injection of LPS, 10 mg per kg body weight, (n = 5 per group) or *E. coli* (**C**), i.p. infection with 1 × 10^7^ *E. coli 0111:B4*, n = 5 per group). Alternatively, CLP was performed (**D**), n = 5, per group) or colitis was induced by 3.0% DSS (E, n = 5, per group) followed by i.p. injection with MS (16 ml/kg body weight) or saline. 6 h (**B,C**) or 7 days (**F**) later, the blood sera were collected and the levels of TNF-α and IL-6 were measured by ELISA. The survival of CLP mice receiving isotype antibody or anti-IL-10 antibody mice was monitored at the indicated time every day (**D**), n = 8, *P < 0.05 Wilcoxon test. The weights and disease activity indices of DSS-induced mice were monitored every day. The results in (**A–F**) are presented as the mean ± SD. *P < 0.05; **P < 0.01; ***P < 0.001 (Student’s t-test).

To investigate whether the enhancement of IL-10 production account for the decreased production of TNF-α and IL-6 observed in MS-treated macrophages following LPS stimulation, We analysed the TNF-α and IL-6 levels in LPS-stimulated macrophages in the presence of anti-IL-10 antibody. Neutralization of IL-10 restored the amount of pro-inflammatory cytokines in a dose-dependent manner (Fig. 6A). In the presence of 10 μg/ml anti-IL-10 antibody, the anti-inflammatory activity of MS was almost completely abrogated in macrophages (Fig. 6A).

Next we investigated the role of IL-10 in endotoxin shock mice. Mice were intraperitoneally injected with LPS alone or in combination with anti-IL-10 antibody or isotype antibody. As shown in Fig. 6B, the presence of anti-IL-10 antibody nearly completely abrogated the inhibitory effect of MS, resulting in an almost complete restoration of the levels of both TNF-α and IL-6, supporting the crucial role of IL-10 in endotoxin shock mice. Similar results were obtained from bacterially challenged mice (Fig. 6C). The neutralization of IL-10 reversed the decreased production of TNF-α and IL-6 in MS-treated CLP mice. Accordingly, CLP mice that neutralized IL-10 had a significantly lower survival rate compared with mice receiving the isotype control antibody (Fig. 6D).

We further examined the contribution of IL-10 to the anti-inflammatory effect of MS in the DSS-induced colitis model. We found that the blockade of IL-10 markedly attenuated the inhibitory effect of MS, as shown by exacerbated colon inflammation, exaggerated weight loss, higher DAI and elevated amount of IL-6 (Fig. 6E). The levels of TNF-α were also examined. And the TNF-α levels in the serum on day 8 of DSS treatment were undetectable by ELISA (data not shown). These *in vitro* and *in vivo* evidence supports that IL-10 production is required for the protective effect of MS in the innate inflammatory response.

### IL-10 contributed to the attenuation of TLR4-induced NF-κb/MAPKs activation

**Figure 7:**
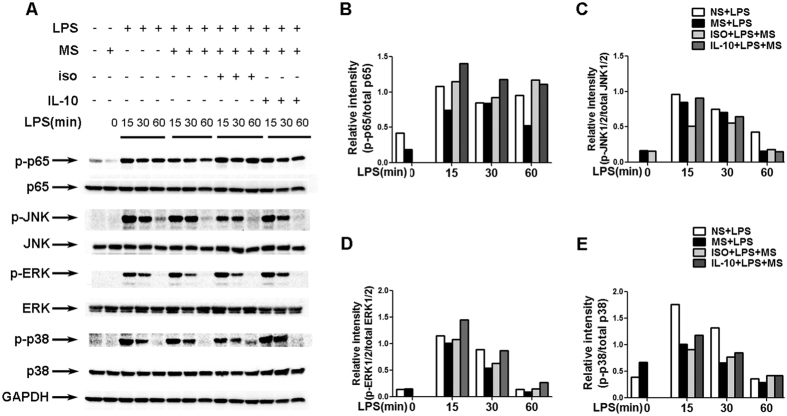
Blockade of IL-10 reverses the attenuated LPS-induced activation of NF-κb and MAPKs in MS-treated macrophages. (**A**) Peritoneal macrophages were pre-treated with 5 μg/ ml isotype or anti-IL-10 antibody for 1 h  , followed by 100 ng/ml LPS stimulation. MS was added 5 min after LPS administration, and the protein levels of the indicated molecules were examined by western blotting. (**B–E**) The results were quantified by determining the band intensity and calculated as the ratio of phosphorylated signalling molecules to total corresponding molecules. The data are representative of at least 2 independent experiments.

We have demonstrated the crucial role of IL-10 in suppressing the pro-inflammatory cytokine production induced by MS upon TLR4 activation both *in vitro* and *in vivo*. Considering the observation that MS can suppress TLR4-induced NF-κb/MAPKs activation (Fig. 4), we next determined whether this attenuated activation of NF-κb/MAPKs is also dependent on IL-10 production. Consistent with the *in vivo* findings, IL-10 blockade 1 h before LPS stimulation at least partially reversed the effects of MS on the decreased phosphorylation of NF-κb p65 and P38MAPK (Fig. 7). Moreover, the blockade of anti-IL-10 antibodies almost completely restored the attenuated activation of JNK and ERK mediated by MS after LPS stimulation, suggesting that IL-10 exert its inhibitory effect differently on MAPKs signalling molecules. These data indicate that IL-10 may be responsible for the inhibition of TLR4-triggered activation of the NF-κb/MAPKs pathways in MS-treated macrophages, which contributes to the reduced production of pro-inflammatory cytokines induced by LPS.

### GSK-3-mediated IL-10 expression via the PI3K-AKT pathway contributes to the inhibitory effect of MS

The TLR-mediated induction of IL-10 transcription in macrophages requires signalling through MAPK, ERK, and P38MAPK^25^,^26^. However, we did not observe enhanced levels of phosphorylation of P38MAPK, ERK and JNK in MS-treated macrophages, suggesting that the signalling pathway of MS induced IL-10 production is independent of MAPKs.

**Figure 8:**
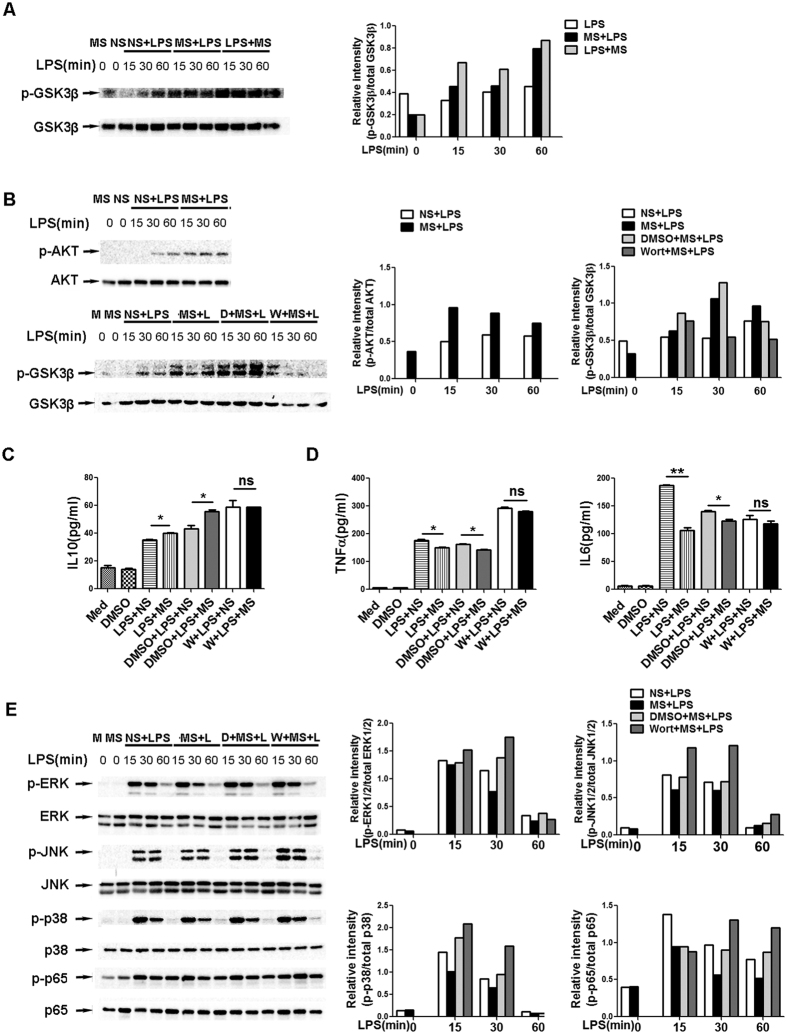
GSK-3β-mediated IL-10 expression via the activation of PI3K-AKT contributes to inhibitory effect of MS. (**A,B**) Peritoneal macrophages were treated with 100 ng/ml LPS alone or in combination with MS (10 μl/ml). The levels of phosphorylation of GSK-3β (**A**) at Ser9 and AKT (**B**), upper figure) at the indicated time points were examined by western blotting. For PI3K inhibition (**B**) lower figure, (**C,E**), PMs were pre-treated with 1 μM wortmannin for 15 min, followed by MS (10 μl/ml) for another 15 min and then stimulated with LPS (100 ng/ml). The supernatants were collected 6 h after LPS stimulation to analyse the level of IL-10 (**C**), TNF-α and IL-6 (**D**) by ELISA. (**E**) The protein levels of the indicated molecules at the indicated time points were examined by western blotting. The results in (**A,B,E**) were quantified by determining the band intensity and calculated as the ratio of phosphorylated signalling molecules to total corresponding molecules. The data are representative of at least 2 independent experiments.

Previous studies have revealed that GSK-3β, a serine/threonine kinase, plays an important role in regulating IL-10 expression^27^,^28^. We therefore investigated whether GSK-3β was involved in MS-induced IL-10 production. The levels of GSK-3β phosphorylation (inactivation) was compared between MS pre-treatment, post-treatment group and control group upon TLR4 stimulation in macrophages. As shown in Fig. 8A, TLR4 signalling led to the enhancement of GSK-3β phosphorylation, and both MS pre-treatment and MS post-treatment induced significantly increased levels of GSK-3β phosphorylation at Ser9 (the major GSK-3β regulating site) compared with the NS control. These data suggest the possibility that increased levels of p–GSK3β at Ser9 may be involved in the regulation of IL-10 production in MS-treated macrophages.

TLR signalling can induce the phosphorylation of GSK-3β through the PI3K-Akt–signalling pathway^29^. To determine whether MS induces GSK-3β phosphorylation via this pathway, we first determined the phosphorylation status of AKT in NS or MS-treated macrophages in the presence of LPS. We found that MS indeed induced elevated levels of p-AKT at all examined time points compared with the NS control(Fig. 8B), indicating that PI3K-Akt may be involved in the MS-mediated activation of GSK-3β. Next, we wondered whether the signalling blockade of PI3K-Akt diminishes GSK-3β phosphorylation. Wortmannin, a specific inhibitor of the PI3K pathway, was used before MS pre-treatment, and p–GSK-3β was analysed by western blotting at the indicated time point. Our data showed that wortmannin inhibited LPS–induced GSK-3β phosphorylation at all examined time points, compared with the DMSO control (Fig. 8B). Accordingly, the MS-induced IL-10 level was reduced (Fig. 8C), and TNF-α and IL-6 production were restored (Fig. 8D). These results strongly indicate that the inhibition of the PI3K-Akt pathway can abrogate the MS-mediated anti-inflammatory effect in LPS-stimulated macrophages through decreased IL-10 production via attenuated GSK-3β phosphorylation.

Finally, we investigated whether PI3K inhibition could attenuate the repressive effect of MS on the LPS-induced activation of NF-κb/MAPKs, which contributed to the decreased production of pro-inflammatory cytokines. Western blot analysis showed that compared with the DMSO control, the downregulation of NF-κb p65, JNK, ERK and P38MAPK activation were at least partially restored by wortmannin in MS-treated macrophages following LPS stimulation (Fig. 8E). These data support the idea that the GSK-3β phosphorylation induced by MS is channelled through a signalling pathway dependent on PI3K-Akt, which contributes to the production of IL-10 and subsequently suppresses the LPS-induced activation of NF-κb and MAPK, ultimately limiting the TLR4-triggered innate immune response.

## Discussion

Previous studies of MS have focused on its protective effect on ischaemia-reperfusion injury or diabetic retinopathy in rats^18^,^19^,^20^,^21^,^22^. Here, we first show that MS can control inflammatory responses that are mainly mediated by the innate immune system, such as endotoxic shock, bacteria-induced sepsis and DSS-induced colitis, in mice via the suppression of pro-inflammatory cytokine production. Mechanistically, MS treatment significantly increased the production of IL-10, which contributed to the attenuated phosphorylation of NF-κB, JNK, ERK and P38MAPK in LPS-stimulated macrophages. The neutralization of IL-10 abrogated the inhibitory effect of MS both in macrophages and in endotoxic shock, bacteria-induced sepsis, CLP and DSS-induced colitis models, suggesting the requirement for IL-10 in the protective effect of MS. The enhancement of GSK-3β phosphorylation (inactivation) via PI3K-AKT was involved in the MS-induced production of IL-10.

Although it is non-toxic, methane is extremely flammable and explosive when it mixes with air. The 2.5% methane gas used by Boros *et al*. was unsafe for administration^13^. In our study, methane was dissolved in normal saline under high pressure, as previously reported^19^,^20^,^22^. It has been demonstrated that the concentration of methane remained relatively stable over one month and can be used for therapeutic purposes^19^,^20^. This methane-rich saline has shown promising protective effects in liver injury^20^, intestine injury^18^, and myocardial injury^19^ induced by ischaemia-reperfusion in rats or dogs. In our *in vivo* study, MS was given intraperitoneally. We measured the methane concentration in the blood after i.p. MS treatment using gas chromatography and found that the peak serum concentration of methane occurred as early as 10 min after i.p. administration and disappeared after approximately 90 minutes (data not shown). We are surprised that the methane level in the blood reached its peak concentration and disappeared so rapidly; we believe that this effect may have contributed to the better effect of MS post-treatment than that of pre-treatment. Our data established that MS treatment significantly improved the survival of mice with endotoxic shock and CLP mice and attenuated bacteria-induced sepsis and DSS-induced colitis. These findings could be attributed to the reduced production of TNF-α and IL-6, which play an important role in the pathogenesis of these models. Notably, our *in vivo* study was performed in animal models after LPS, bacteria, CLP or DSS challenge, revealing the potential value of MS as a therapy to ameliorate the innate inflammatory response. We did not investigate the possible role of MS in other systemic inflammatory diseases, such as RA and SLE. Given the importance of TNF-α and IL-6 in the pathogenesis of autoimmune disease, we infer that MS might also exert a protective effect on RA and SLE. Our study provides a novel and promising strategy for the treatment of inflammatory immune response-mediated diseases such as endotoxic shock, bacteria-induced sepsis, CLP and DSS-induced colitis mice, expanding the scope of the therapeutic applications of methane.

It has been reported that MS could protect against ischaemia-reperfusion injury through its anti-oxidative, anti-apoptotic and anti-inflammatory effects^19^,^20^,^22^. Although the levels of TNF-α and IL-1β were reduced by MS treatment in ischaemia-reperfusion injury, it remains unclear how methane inhibits inflammatory cytokine production. Our data first established that MS suppresses the production of the pro-inflammatory cytokines TNF-α and IL-6 at the mRNA level as well as at the protein level, due to the attenuated activation of NF-κb and P38MAPK in macrophages. Notably, MS inhibited IL-6 production more significantly than TNF-α, especially at the mRNA level, in mouse peritoneal macrophages, which is consistent with our *in vivo* results of the DSS colitis model, which showed that MS treatment reduced the production of IL-6 by 67–90% compared with control mice. This result suggests that MS may exert differential degrees of inhibition on different cytokines depending on the cell type and the animal model used. Whether MS inhibits the production of other cytokines or chemokines, such as IL-1β, MIP-2, IL-12p40 or type I interferons, requires further investigation.

Our study demonstrated the requirement of IL-10 in the MS-mediated protective effect in the TLR4-stimulated immune response both *in vitro* and *in vivo*. Blockade with anti-IL-10 antibody reversed the MS-mediated decreased production of TNF-α and IL-6 in LPS-simulated macrophages. In our *in vivo* models, neutralization of IL-10 abrogated the protective effect of MS in mice with endotoxin shock or bacteria-induced sepsis and CLP mice as well as in mice with DSS-induced colitis, further confirming the crucial role of IL-10 in the protective effect of MS. The elevated level of IL-10 attenuated the LPS-induced activation of NF-κB, JNK, ERK and P38MAPK, which resulted in the reduced production of TNF-α and IL-6. We do not know whether MS induced IL-10 production in other immune cells, such as CD4^+^ T cells or neutrophils. So, it is possible that IL-10 derived from other immune cells may be involved in the MS-mediated anti-inflammatory effect. It is also possible that MS exerted its protective effect through its anti-oxidative, anti-apoptotic mechanisms in these animal models of our studies. Given the observation that IL-10 blockade almost completely abrogates the protective effect of MS *in vivo*, we believe that the IL-10 pathway may play more important roles in the inhibitory effect of MS in our models. Our findings enhance the understanding of the anti-inflammatory mechanism of MS.

IL-10 is produced by many cells of the immune system, including macrophages and T cells^24^,^30^. The mechanism that controls IL-10 production in macrophages in response to defined stimuli has been shown to involve MAPKs such as ERK and P38MAPK^25^ and transcription factors such as CREB, NF-κB p50 homodimers, and C/EBPb^26^,^31^,^32^,^33^,^34^. However, we did not observe increased activation of ERK, P38MAPK, or CREB (data not shown) in MS-treated macrophages, but provide evidence that MS activates GSK-3β at Ser^9^ via the PI3K/AKT pathway to increase LPS-induced IL-10 production. MS induced the marked enhancement and prolonged expression of p-GSK-3β and p-AKT, and PI3K inhibition attenuated the activation of GSK-3β and reduced IL-10 production, indicating the involvement of the PI3K-AKT-GSK-3β pathway in MS-induced IL-10 production. Our study was in consistent with previous reports, that the phospho-inactivativation of GSK-3β downregulates TLR-mediated inflammatory responses, while increases IL-10 production^35^,^36^,^37^. It was reported that PI3K signalling acts to downregulate TLR signalling through the induction of IL-10 via mammalian target of rapamycin (mTOR), the activation of GSK-3β, and the phosphorylation of Foxo1^38^,^39^,^40^, so mTOR and Foxo1 may also act as upstream mediators of p-GSK-3β-induced IL-10 production in MS-treated macrophages.

We do not know how MS exerts its effect on the PI3K-AKT pathway. It has been hypothesized that methane might act on membrane channels such as G-proteins, membrane receptor-mediated signalling, and acetylcholine-activated ion channel kinetics^41^,^42^,^43^, or methane might accumulate transiently at the interfaces of cell membranes, affecting the function of membrane-bound enzymes^12^. The activation of GSK-3β via PI3K-AKT by MS occurs extremel rapid, that upregulation of IL-10 mRNA expression was observed only 15 min after MS exposure in LPS-stimulated macrophages in our study. Considering that methane is the simplest aliphatic hydrocarbon with a high gaseous-diffusion coefficient, only 10 min was required to reach the peak blood concentration after administration of MS, we infer that methane could easily penetrates membranes and diffuses into nucleus and activating GSK-3β^11^,^44^. All the above properties of MS may explain its rapid and effective anti-inflammatory responses.

It has been shown that phospho-inactivativation of GSK-3β suppresses MyD88-dependent cytokine production but upregulates IL-10 production in innate immune cells exposed to bacterial stimuli^27^,^31^,^36^. However, the molecular mechanisms underlying how GSK-3β differentially regulates the TLR-mediated production of pro- and anti-inflammatory cytokines are poorly characterized. Active GSK-3β is believed to attenuate NF-κb activation by enhancing interactions between CREB with the CBP, which attenuates CBP binding with NF-κb^36^. Alternatively, GSK-3β may facilitate NF-κb activity by activating NF-κb p65^45^ and limiting NF-κb activation via as yet unidentified pathways^46^. Indeed, we did observe the MS-mediated enhancement of GSK-3β activation and attenuated activation of NF-κb/MAPKs, which is associated with the limited production of pro-inflammatory cytokines triggered by TLR ligation. It might be possible that the activation of GSK-3β inhibited the activation of NF-κb via the above-mentioned pathway, but it is more likely that the attenuated activation of NF-κb/MAPKs were due to GSK-3β-mediated IL-10 production in our study because the neutralization of IL-10 resulted in at least a partial reversal of the decreased activation of NF-κb and MAPKs. In particular, *in vivo* blockade of IL-10 led to nearly complete abrogation of the protective effect of MS, which further emphasized the crucial role of IL-10 mediated by GSK-3β in suppressing the TLR-triggered signalling pathway. One of the transcription factors that is negatively regulated by GSK-3b is CREB, which positively regulates IL-10 expression, but we did not observe the upregulation of CREB (data not shown). A recent study showed that GSK-3β could regulate IL-10 expression in CD4^+^ T cells through epigenetic modification of the IL-10 promoter^47^. Given that the intracellular signalling pathways that control IL-10 production are complex and may vary depending on the cell type and stimuli, how p-GSK-3β regulates IL-10 expression has not been resolved in our study and requires further investigation.

## Conclusion

Here, we described a novel role of and mechanism for MS in suppressing inflammation in mouse macrophages and in four examined mouse models: the endotoxin shock model, bacteria-induced sepsis model, CLP and DSS-induced acute colitis model. Mechanistically, beyond the intrinsic role of MS’s anti-oxidative, anti-apoptotic and anti-inflammatory effects reported in ischaemia-reperfusion injury models, we found that treatment with MS resulted in a significantly lower level of TNF-α and IL-6 as well as an elevated level of IL-10 upon LPS exposure in macrophages and in animal models. Blockade of IL-10 with anti-IL-10 antibody almost completely abrogated the anti-inflammatory activity of MS both *in vitro* and *in vivo*, which emphasized the requirement for IL-10 in the protective effect of MS. We also provided further evidence that the phospho-inactivation of GSK-3β via enhanced PI3K/AKT signalling was involved in the increased IL-10 production mediated by MS, which led to the attenuated phosphorylation of NF-κb and MAPKs and resulted in the downregulation of pro-inflammatory cytokines and repressed inflammatory responses both *in vitro* and *in vivo*. Our data improve our understanding of the mechanism underlying MS-mediated repression of the immune response and suggest that MS may represent a novel therapeutic approach.

## Methods

### Animals

C57BL/6J mice were obtained from Shanghai SLAC Laboratory Animals (Shanghai, China). All animal experiments were performed in accordance with the National Institutes of Health Guide for the Care and Use of Laboratory Animals with the approval of the Scientific Investigation Board of Second Military Medical University.

### Cell culture

Thioglycollate-elicited mouse peritoneal macrophages were prepared from C57BL/6J mice (6–8 weeks old) and cultured in RPMI 1640 with 10% (v/v) FCS. Bone marrow–derived macrophages were generated in RPMI 1640 medium with recombinant mouse M-CSF (10 ng/ml) as described previously^48^. Cells (3 × 10^5^) were seeded into 24-well plates for cytokine assays, and 2 × 10^6^ cells were seeded into 6-well plates for immunoblot analysis.

### Preparation of methane-rich saline

Methane-rich saline (MS) was provided by the Department of Diving Medicine, Faculty of Navy Medicine, Second Military Medical University (Shanghai, China). To obtain a supersaturated level of methane, methane was dissolved in physiological saline for 6 h under high pressure (0.4 MPa). The concentration of methane in the saline was measured using gas chromatography, as described by Oshawa *et al*.^49^. The concentration of the methane-rich saline was 0.99 mmol/L using methane-containing standard gas (Shanghai Jiliang Standard Gas Ltd., Shanghai, China) for comparison. MS was freshly prepared one day before the animal experiments to ensure a steady concentration in the injection and stored under atmospheric pressure at 4 °C for 24 h.

### Animal models of sepsis

Endotoxaemia (endotoxic shock) was induced by the i.p. injection of 10 mg/kg LPS. For endotoxaemia survival experiments, the mice were i.p. injected with 15 mg/kg LPS. For bacteraemia induction (bacterial challenge), the mice were i.p. injected with 1 × 10^7^ cfu of Gram-negative *E. coli*. (ATTC 25922). Experimental sepsis was induced by caecal ligation and puncture, and the survival of the mice was monitored.

### Bacterial load determination

Mice were infected with *E. coli* i.p and euthanized 5 h post-infection. The blood was collected, serially diluted in sterile PBS, and plated onto separate Luria-Bertani agar plates, and the bacterial recovery was determined by counting colonies after overnight incubation.

### DSS-induced colitis

Dextran sodium sulfate (DSS, m.w. 36,000; MP Biomedicals) was administered ad libitum in distilled water at a 3% concentration for 5 d followed by normal drinking water. Fresh DSS solution was prepared daily. The mice were divided into groups of 5–7 mice and were sacrificed at the indicated time points. In the control group (day 0), the animals received only water. Body mass, fur texture, posture, stool consistency and faecal bleeding were recorded daily. The disease activity index (DAI) comprised fur texture, animal posture and stool consistency.

### Treatment with methane-rich saline

The dose and timing of MS treatment in the dose-response study of peritoneal macrophages was as indicated in Fig. 1. For the *in vitro* experiments, peritoneal macrophages were treated with MS (10 μl/ml) or NS at the indicated time points after LPS stimulation. In the mouse experiments^20^, the peak plasma level of MS occurred 10 min after i.p administration. Our research team member investigated the effective dosage of MS in another study of LPS-induced lung injury of mice (data not shown). Briefly, 8 ml/kg MS did not elicit a protective effect, and there was no difference between the effects of the 16 ml/kg and 25 ml/kg MS doses. Furthermore, it is reported that the volume resuscitation effect of the i.p. administration of 35 ml/kg MS may alleviate inflammation in mice^50^. Hence, 16 ml/kg MS was chosen in our *in vivo* experiment. The mice were treated with 16 ml/kg MS or saline 30 min after LPS or bacterial challenge in septic animal models. In the DSS-induced colitis model, the mice were given 16 ml/kg saline or MS i.p. on day 2, day 3 and day 4 during DSS administration.

### Anti-IL-10 antibody administration

The dose of anti-IL-10 antibody administration in the dose-response study of peritoneal macrophages was from 2 μg/ml, 10 μg/ml to 20 μg/ml. For the *in vitro* experiments, peritoneal macrophages were treated with anti-IL-10 antibody (5 μg/ml) or isotype 1 h before LPS stimulation. Anti-IL-10 antibody or isotype was i.p. injected 40 μg/per mouse 24 h before LPS or bacterial challenge in septic animal models. For the DSS-induced colitis model, anti-IL-10 antibody or isotype was i.p. injected 40 μg/per mouse 24 h before DSS administration.

### Histology

Colons were removed and 3 cm of the distal colon was fixed in 4% paraformaldehyde solution for at least 48 h before being embedded in paraffin and sectioned (6 μm). After deparaffinization and rehydration, the sections were stained with haematoxylin and eosin. Histologic changes were evaluated by two pathologists who were blinded to the treatment regimen.

### Cytokine PCR

Total cellular RNA was extracted using the Trizol reagent according to the manufacturer’s instructions (TaKaRa). Single-strand cDNA synthesis was performed using a PrimeScript RT reagent kit (TaKaRa). Target gene expression was normalized to the reference gene, GAPDH. The 2−ΔΔCt method was applied to calculate the relative gene expression. The PCR products were subjected to melting curve analysis and a standard curve to confirm the correct amplification. All the RT-PCRs were performed in triplicate.

### Cytokine ELISA

For cytokine ELISA, supernatants from cultured cells were collected at different time points. The total IL-10, IL-6 and TNF-α were measured by enzyme-linked immunosorbent assay (ELISA) with ELISA kits (R&D system Minneapolis, MN) according to the manufacturer’s protocol.

### Western blot analysis

Immunoblotting was performed according to standard methods. Briefly, after extraction, the proteins in the cell lysates were first resolved by SDS-polyacrylamide gel electrophoresis and then transferred to PVDF membranes and subsequently incubated with primary antibodies. After incubation with peroxidase-conjugated secondary antibodies, the signals were visualized by enhanced chemiluminescence (Pierce, Rockford, IL, USA, # 32106) according to the manufacturer’s instructions. The relative band intensity was quantified using Image J 1.47v. The antibodies and reagents are listed in Table S1.

### Statistical analysis

The Prism 5 software package (GraphPad) was used to calculate the *p* values using an unpaired two-tailed Student’s t test, one-sample t test, one-way ANOVA, or Wilcoxon test. *p* values < 0.05 were considered statistically significant.

## Additional Information

**How to cite this article**: Zhang, X. *et al*. Methane limit LPS-induced NF-κB/ MAPKs signal in macrophages and suppress immune response in mice by enhancing PI3K/AKT/GSK3β-mediated IL-10 expression. *Sci. Rep*. **6**, 29359; doi: 10.1038/srep29359 (2016).

## Supplementary Material

Supplementary Information
