# Supplementary material for: Methane limit LPS-induced NF-κB/MAPKs signal in macrophages and suppress immune response in mice by enhancing PI3K/AKT/GSK-3β-mediated IL-10 expression
Source: Sci Rep. 2016 Jul 11;6:29359. doi: 10.1038/srep29359 (PMC4942692; doi:10.1038/srep29359)
Supplement: Supplementary Information [file srep29359-s1.doc]

**Methane limit LPS-induced NF-κB/ MAPKs signal in macrophages and suppress immune response in mice by enhancing PI3K/AKT/GSK-3β-mediated IL-10 expression**

Xu Zhang^1,2,3^, Na Li^1^, Han Shao^1,2,3^, Yan Meng^1^, Liping Wang^1,4^, Qian Wu^1^, Ying Yao^1^, Jinbao Li^1^, Jinjun Bian^1^, Yan Zhang^1^,*****, Xiaoming Deng^1^,*****

Table S1

Name Cat. no./vendor

Reagent:

LPS 0111:B4/Sigma-Aldrich

DSS 02160110/MP Biomedicals

M-CSF AF-315-02/PeproTech

Anti IL10 16-7101/eBioscience

Iso-type 16-4031/eBioscience

Wortmannin 9951S/Cell Signaling

Antibody:

Erk 9102S/Cell Signaling

p-ERK 9106S/Cell Signaling

JNK 9258S/Cell Signaling

p-JNK 9255S/Cell Signaling

P38 9212S/Cell Signaling

P-P38 9211S/Cell Signaling

P-P65 93H1/Cell Signaling

P65 8242S/Cell Signaling

GSK3β 9315/Cell Signaling

P-GSK3β 9323/Cell Signaling

AKT C67E7/Cell Signaling

P-AKT S473/Cell Signaling

GAPDH 14C10/Cell Signaling
